# Supplementary material for: Dithionite quenching of NBD-labeled lipids reveals artificial lipid droplet purity and neutral lipid surface accessibility
Source: J Lipid Res. 2026 Jul 17;67(8):101105. doi: 10.1016/j.jlr.2026.101105 (PMC13495605; doi:10.1016/j.jlr.2026.101105)
Supplement: Supplementary file 1 [file mmc1.pdf]

Supplemental Information

Dithionite quenching of NBD-labeled lipids reveals artificial lipid droplet purity and neutral lipid surface accessibility

Jiyao Chai[1], Lingshuang Wu[1], Yong Mi Choi[1], Shujuan Gao[1], Daniel Canals[2], Abdou Rachid Thiam[3], Erwin London[1], Michael V. Airola[1*]

[1] Department of Biochemistry and Cell Biology, Stony Brook University, Stony Brook NY 11794, USA

[2] Department of Medicine, Renaissance School of Medicine at Stony Brook University, Stony Brook NY 11794

[3] Laboratoire de Physique de l'École normale supérieure, ENS, Université PSL, CNRS, Sorbonne Université, Université Paris Cité, F-75005, Paris, France

*Address correspondence to:

Michael V. Airola, michael.airola@stonybrook.edu

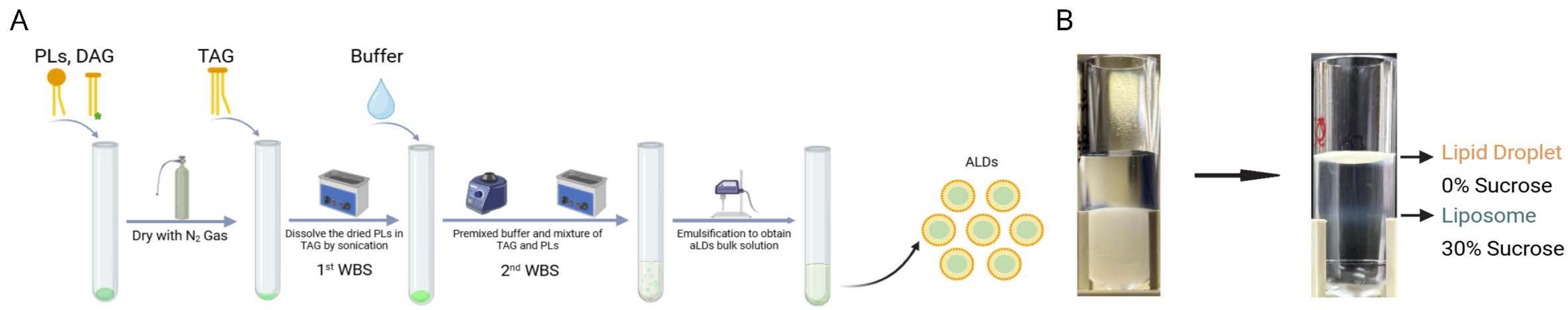

Supplementary Figure 1. Artificial lipid droplet preparation and sucrose centrifugation.

(A) Schematic of the original artificial lipid droplet preparation protocol. Phospholipids and fluorescently labeled neutral lipids were dried under nitrogen in a glass test tube to form a thin lipid film. Triolein was added and mixed by water-bath sonication (1st WBS). HN buffer was then introduced, followed by brief vortexing and a second water-bath sonication (2nd WBS) step. The mixture was finally emulsified by microtip sonication to generate the bulk artificial lipid droplet solution. **(B)** Sucrose centrifugation leads to a purified aLD layer (top), and liposome layer (middle).

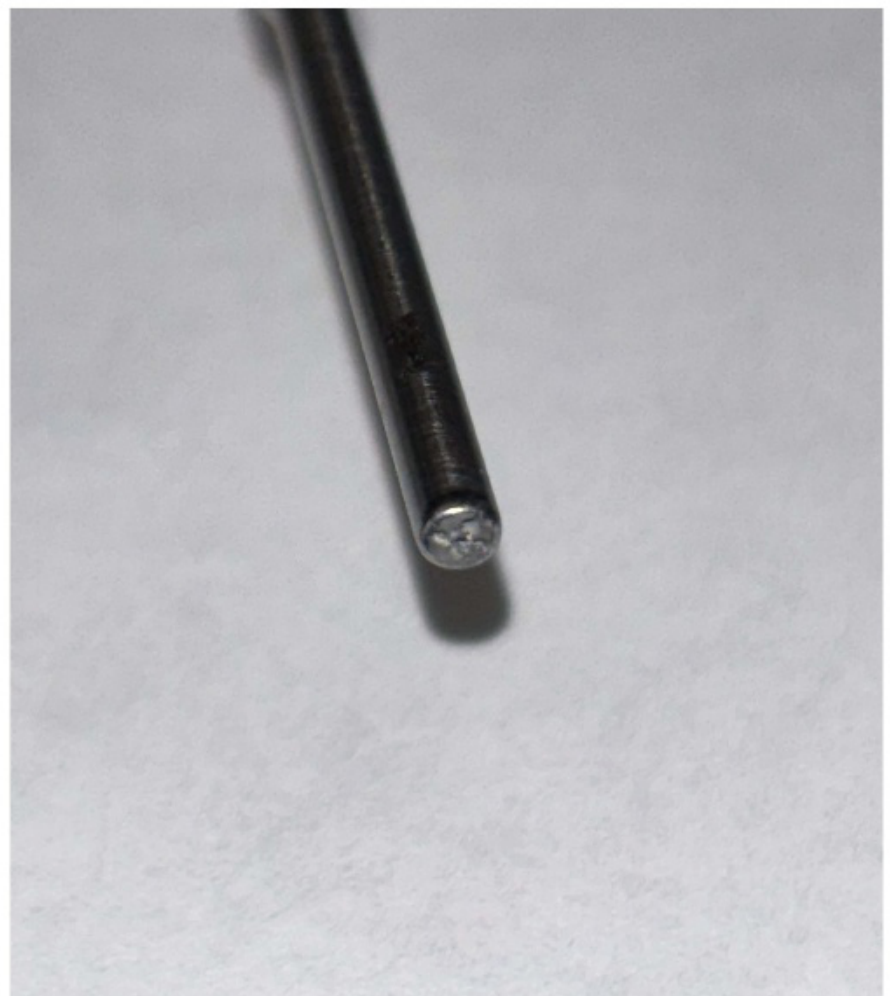

Supplementary Figure 2. Sonicator Micro-tip.

Picture of the titanium probe and micro-tip used in this study: a Fisher scientific 1/8 in. (3.2mm) Probe (13.8cm x 1.3cm) for processing 0.5-15 mL volumes (catalog# FB4422).

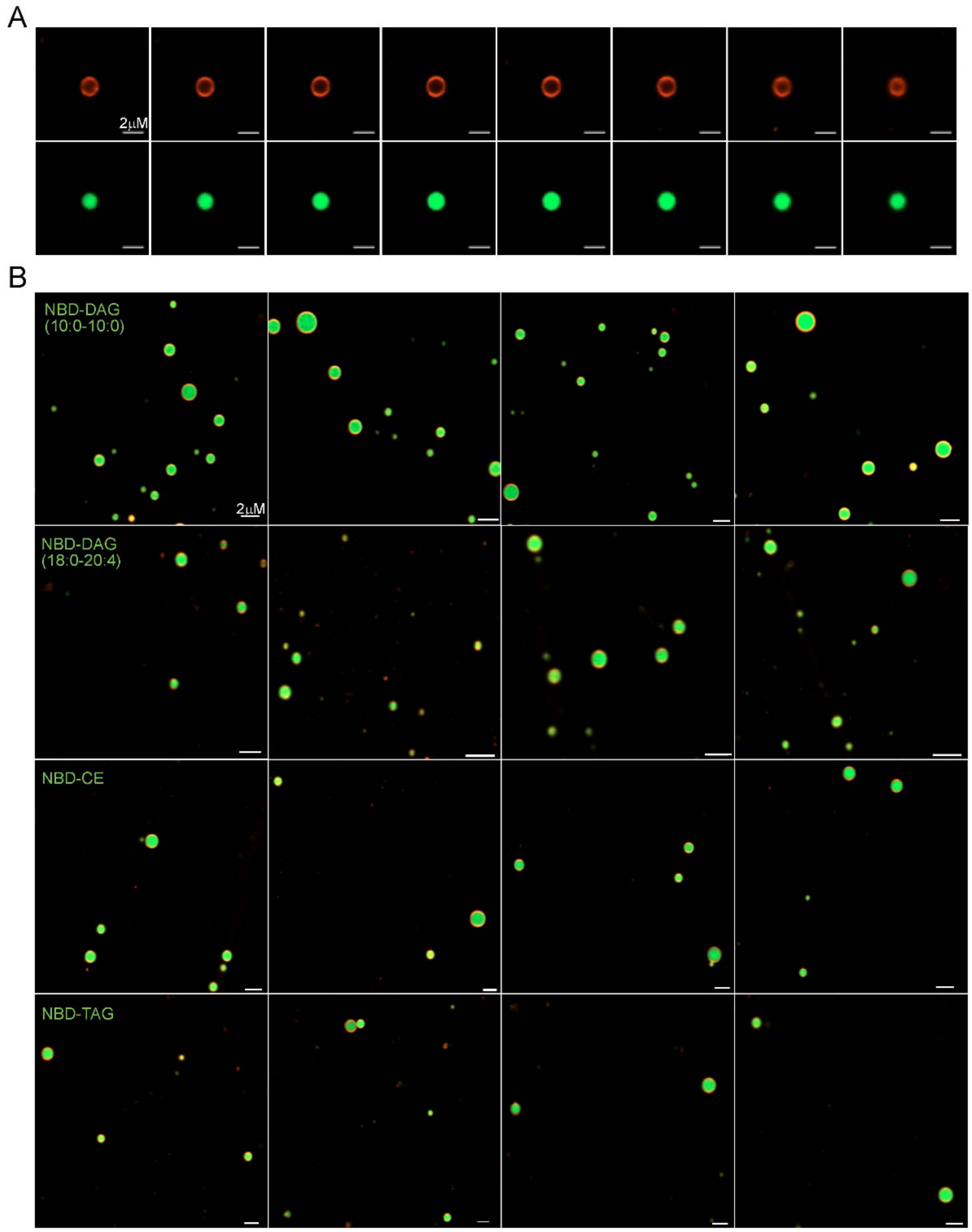

Supplementary Figure 3. Z-stack image for a single aLD.

(A) Z-stack confocal fluorescence imaging of the representative aLD shown in Fig. 6B. The droplet contains NBD-DAG (10:0–10:0) as the fluorescent neutral lipid probe. Rhodamine-PE, shown in red, marks the phospholipid monolayer, while NBD-DAG, shown in green, reports the distribution of the labeled neutral lipid. Optical sections were acquired across the z-axis of the droplet at 0.17 μm intervals between adjacent planes. Scale bar = 2 μm. **(B)** Confocal fluorescence imaging for artificial lipid droplets labeled with NBD-DAG (10:0-10:0), NBD-DAG (18:0-20:4), NBD-CE, or NBD-TAG (green) and rhodamine-PE (red). Scale bar = 2 μm.
